# A Cytohesin Homolog in *Dictyostelium* Amoebae

**DOI:** 10.1371/journal.pone.0009378

**Published:** 2010-02-23

**Authors:** Maria Christina Shina, Rolf Müller, Rosemarie Blau-Wasser, Gernot Glöckner, Michael Schleicher, Ludwig Eichinger, Angelika A. Noegel, Waldemar Kolanus

**Affiliations:** 1 Center for Biochemistry, Medical Faculty, Center for Molecular Medicine Cologne (CMMC) and Cologne Excellence Cluster on Cellular Stress Responses in Aging-Associated Diseases (CECAD), University of Cologne, Köln, Germany; 2 Leibniz Institute for Age Research - Fritz-Lipmann-Institute e.V., Jena, Germany; 3 Institute of Anatomy and Cell Biology and Center for Integrated Protein Science (CIPSM), Ludwig-Maximilians-University, Muenchen, Germany; 4 Laboratory of Molecular Immunology, LIMES Institute of the University of Bonn, Bonn, Germany; CNRS UMR6543, Université de Nice, Sophia Antipolis, France

## Abstract

**Background:**

*Dictyostelium*, an amoeboid motile cell, harbors several paralogous Sec7 genes that encode members of three distinct subfamilies of the Sec7 superfamily of Guanine nucleotide exchange factors. Among them are proteins of the GBF/BIG family present in all eukaryotes. The third subfamily represented with three members in *D. discoideum* is the cytohesin family that has been thought to be metazoan specific. Cytohesins are characterized by a Sec7 PH tandem domain and have roles in cell adhesion and migration.

**Principal Findings:**

*Dictyostelium* SecG exhibits highest homologies to the cytohesins. It harbors at its amino terminus several ankyrin repeats that are followed by the Sec7 PH tandem domain. Mutants lacking SecG show reduced cell-substratum adhesion whereas cell-cell adhesion that is important for development is not affected. Accordingly, multicellular development proceeds normally in the mutant. During chemotaxis *secG^−^* cells elongate and migrate in a directed fashion towards cAMP, however speed is moderately reduced.

**Significance:**

The data indicate that SecG is a relevant factor for cell-substrate adhesion and reveal the basic function of a cytohesin in a lower eukaryote.

## Introduction

Cell motility and transient cell contacts are essential during mammalian development and for cells of the immune system. Both aspects are mediated by integrins, heterodimeric cell surface receptors that bind to the extracellular matrix and have roles in intercellular adhesion as well. In the cytosol a number of integrin interacting proteins are known which influence the conformation and activity of integrins such as cytoskeletal, adaptor and signaling proteins [1]. One of them, the guanine nucleotide exchange factor cytohesin-1 activates beta-2 integrin in lymphocytes involving RhoA [2]. Overexpression of cytohesin-1 in Jurkat T cells results in upregulation of LFA-1 (leukocyte specific beta-2 integrin) adhesion to ICAM-1 whereas knockdown of cytohesin-1 reduced the adhesion [3]. The knockdown further resulted in migration defects and in reduced chemotaxis in bone marrow-derived dentritic cells suggesting an important role of cytohesin in amoeboid migration [2].

Cytohesin-1 is a member of a larger group of proteins characterized by the presence of PH domains and a GEF domain. Based on the homology of their GEF domain to the one of Arf GEF sec7p of yeast, this group of proteins belongs to the Sec7 family of ArfGEFs.

ArfGEFs catalyze the switch from the GDP- to the GTP-bound form of ArfGTPases. This is crucial for the function of the ArfGTPases as key regulators of vesicular transport in eukaryotic cells, their roles in the regulation of actin cytoskeleton dynamics, cell adhesion and cell migration and in signal transduction processes [4] [5]. Five subfamilies of Sec7 ArfGEFs have been defined that differ with regard to their overall structure and domain composition; the GBF and BIG family, the cytohesin, EFA6, BRAG and FBX family. GBF and BIG type ArfGEFs are involved in Golgi traffic and have been found in all eukaryotes. GBF/BIG proteins are the only Sec7 members present in plants, whereas the cytohesin, EFA6, BRAG and FBX families were only reported for metazoans so far. Yeasts harbour representatives of the GBF/BIG family, and two more proteins which have been thought to be group specific. One of them was recently re-classified as an EFA6 family member [6]. The bacterial pathogens *Rickettsia* and *Legionella* also express ArfGEFs that they may have acquired by gene transfer from their hosts [7]. In the cytohesin, EFA6 and BRAG family, the Sec7 domain is followed by a pleckstrin homology (PH) domain. The structure of the Sec7 domain/Arf complex has been solved and the catalytic mechanism elucidated [8] [9]. The Sec7-domain encompasses 200 amino acids containing 10 alpha-helices that form two subdomains. For the phosphoinositide-dependent cytohesin-1 the crystal structure of the Sec7-PH tandem revealed an autoregulation through the interaction of a C-terminal amphipathic helix with a linker region between the domains [10].

*Dictyostelium discoideum* is an amoeboid motile cell which feeds on bacteria and yeast in the wild. Based on its life style it can be considered a professional phagocyte. Upon starvation cells aggregate by chemotaxis and form a multicellular organism that undergoes a developmental program ending with fruiting body formation. Its genome encodes one ARF, five ARF-like proteins and 10 ARF-related proteins [11]. The number of ArfGEFs is lower and only six ArfGEFs (seven in strains AX3 and AX4 due to a large duplication in chromosome 2) are present. The proteins belong to the GBF, the BIG and the cytohesin family. Here we present an analysis of Sec7 domain proteins in the *Dictyostelia.* We focus on SecG of *D. discoideum*, the closest homolog of the cytohesins in *D. discoideum*, and investigate its function in cell adhesion and cell migration.

## Results

### Sec7 Members in the Genomes of *Dictyostelia*

We carried out an analysis of the Sec7-domain containing proteins in the genomes of *D. discoideum*, *Dictyostelium falciparum* and *Polysphondylium pallidum*. The three species belong to different taxonomic divisions within the *Dictyostelia*. *D. falciparum* belongs to group 1, the most basic group of *Dictyostelia*, *P. pallidum* is a member of group 2 and *D. discoideum* is a member of group 4, the most evolutionarily distant group. Its members use cAMP as chemoattractant for aggregation whereas in the other groups different compounds are used. Furthermore, the group 4 members form solitary fruiting bodies [12].

The *D. discoideum* genome (strain AX4) encodes seven proteins containing a Sec7 domain (dictyBase, http://dictybase.org/index.html) (Table 1). Four of them belong to the family of large ArfGEFs which are further subdivided into the GBF and BIG type large ArfGEFs. One of the *D. discoideum* large ArfGEFs is a member of the GBF family (DDB_G0290771/DDB0233595), the others belong to the BIG family (DDB_G0290369/DDB0233618, DDB_G0273101/DDB0233619, DDB_G0273831/DDB0233619). The BIG family members DDB_G0273101 and DDB_G0273831 are identical copies and are located in the large duplication of chromosome 2 which is only present in *D. discoideum* strains AX3 and AX4 [13]. The GBF subfamily member has a Sec7 domain, while the three BIG proteins harbour in addition a DUF1981 domain (see below). Mouratou et al. [14] identified five non-catalytic domains common to both subfamilies based on sequence homology. These domains include an N-terminal DCB domain allowing dimerization followed by the Homology Upstream of Sec7 (HUS) domain, the Sec7 domain, and three HDS domains (Homology Downstream of Sec7). The domain structure of the *D. discoideum* GBF and Big proteins largely corresponds to this pattern. In the GBF family member the HDS1 domain is interrupted at around position 100 of the HDS1 motif by an unusually long stretch of amino acids rich in asparagines (position 857 to 985), and HDS2 is rather weakly conserved when compared to the mammalian counterpart (25.4% identity in a 118 amino acids (aa) overlap for DDB_G0290771/DDB0233595). In contrast, in the BIG family members the HDS2 motif is well conserved (50.6% identity in a 158 aa overlap in DDB_G0290369/DDB0233618, and 43.7% identity in a 167 aa overlap in DDB_G0273101/DDB0304853 and DDB_G0273831/DDB0233619, respectively) and encompasses the DUF1981 region of homology (Table 1).

**Table 1:** The Sec7 family of the *Dictyostelia*.

Family	Species	Protein coding gene	Gene ID	Length (aa)	Domains
**GBF**					
	*D. d.*	DDB0233595	DDB_G0290771	1,748	Sec7
	*P. pallidum*		PPL_08838	871	Sec7
	*D. f.*		DFA_05701	1455	Sec7
**BIG**					
	*D. d.*	DDB0233618	DDB_G0290369	1,886	Sec7, DUF1981
	*D. d.*	DDB0304853	DDB_G0290369	2,048	Sec7, DUF1981
	*D. d.*	DDB0233619	DDB_G0273831	2,048	Sec7, DUF1981
	*P. p.*		PPL_04049	1618	Sec7, DUF1981
	*P. p*		PPL_08053	1859	Sec7, DUF1981
	*D. f.*		DFA_04299	1956	Sec7, DUF1981
	*D. f.*		DFA_12170	1766	Sec7, DUF1981
**Cytohesin**					
	*D. d.*	DDB0233591	DDB_G0279241	919	MFS, Sec7, PH
	*D. d.*	DDB0233617	DDB_G0272486	931	Sec7, PH
	*D. d.*	DDB0191439	DDB_G0287459	986	Ankyrin, Sec7, PH
	*P. p.*		PPL_10293	686	TM, Sec7, PH
	*P. p.*		PPL_12526	971	Sec7, PH
	*P. p.*		PPL_05633	1696	Ankyrin, Sec7, PH, Zn^2+^finger
	*D. f.*		DFA_11559	678	DUF2339 Sec7, PH
	*D. f.*		DFA_11731	866	Sec7, PH
	*D. f:*		DFA_04214	962	Ankyrin, Sec7, PH

The Sec7 family members in three taxonomic divisions of the *Dictyostelia* are listed. The *D. discoideum* genome [11] was searched for proteins containing a Sec7 domain (dictyBase, http://dictybase.org/index.html). The families were classified according to Casanova [46]. The domains listed were identified by Blast searches (http://blast.ncbi.nlm.nih.gov/Blast.cgi). The *D. discoideum* (*D. d.*) proteins were then used to search for homologues in the *D. falciparum* (*D. f.*) and *P. pallidum* (*P. p.*) genomes at http://sacgb.fli-leibniz.de/cgi/index.pl. *D. falciparum* belongs to group 1, *P. pallidum* is a member of group 2 and *D. discoideum* (*D. d.*) is a member of group 4. The homology search was done by Blast. DUF1981, DUF2339, domains of unknown function, present in predicted membrane proteins. MFS, Major-Facilitator-Superfamily, group of membrane proteins; TM, transmembrane; DUF1981, domain of unknown function, present in predicted membrane proteins, PH, PH-domain.

Three further proteins carry the Sec7 domain in tandem with a PH domain. Such an arrangement is present in the cytohesins, the EFA6 and BRAGs where they occur in combination with further domains. In BLAST searches these three *D. discoideum* proteins show highest homologies to the Sec7 PH tandem domain of cytohesins. Therefore we tentatively classify them as cytohesin homologs although they lack the typical N-terminal coiled coil domain of the cytohesins [15]. The DDB0233591 protein carries transmembrane domains of the MFS family type in its N-terminal region, DDB0233617 has no discernable domain in its N-terminus, and for the N-terminal region of DDB0191439 (SecG) several Ankyrin repeats are predicted. Of the three *D. discoideum* cytohesin family proteins SecG exhibited the highest homology to mammalian cytohesins in both the Sec7 and the PH domain. An analysis of this protein in a rather simple organism like *D. discoideum* might therefore give insights into the basic functions of mammalian cytohesins.

In *D. falciparum* and *P. pallidum* we identified one GBF family protein and two BIG family proteins (Table 2) (http://sacgb.fli-leibniz.de/cgi/index.pl). DFA_05701 is unique as it is more closely related to GBF proteins from plants such as GNOM from Arabidopsis thaliana (GENE ID: 837958) than to the *D. discoideum* homolog DDB0233595. Like *D. discoideum, D. falciparum* and *P. pallidum* have three cytohesin family proteins (Table 1).

**Table 2:** Analysis of chemotactic cell motility of *secG^−^* cells.

strain	Speed (µm/min)	directionality	direction change (deg)	Persistence (µm/min-deg)	roundness
AX2	10.95+/−2.9	0.83+/−0.13	17.53+/−7.54	3.57+/−1.5	51.72+/−6.72
secG-1	8.47+/−2.89*	0.88 +/−0.06	12.15+/−5.02	3.6+/−1.4	44.73 +/−5.24
secG-5	8.16+/−2.25**	0.91+/−0.05	9.91+/−3.38	5.2+/−1.4	50.18+/−4.57

Time-lapse image series were captured and stored on a computer hard drive at 30-second intervals. Images were taken at magnifications of 10X and 40X every 6 s. The DIAS software was used to trace individual cells along image series and calculate motility parameters. Objects whose speed was <2 µm/min were excluded from the analysis. Speed refers to the speed of the cell's centroid movement along the total path; directionality indicates migration straightness; direction change refers to the number and frequency of turns; persistence is an estimation of movement in the direction of the path; and roundness indicates the cell polarity. Values are mean ± standard deviation of >30 cells from three or more independent experiments. The difference in speed was statistically significant (P value 0.0416* and 0.012**).

**Figure 1:**
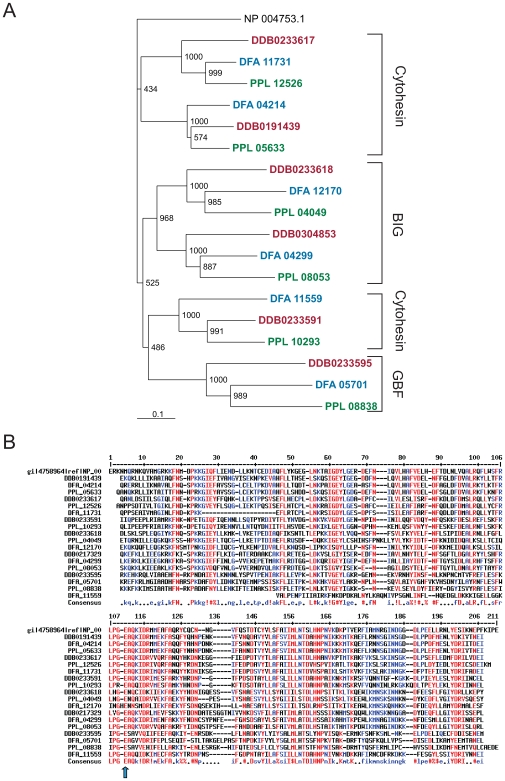
Homology of the Sec7 domain. **A**. Phylogenetic tree of the Sec7 domains in *D. discoideum* (red), *P. pallidum* (green) and *D. fasciculatum* (blue) proteins. The Sec7 domain of human cytohesin-1 (NP_004753, GI:4758964) was used for comparison. The tree was generated from a CLUSTALX alignment with the TreeView program. The assignment of the different proteins to the three subfamilies of the Sec7 family of the Dictyostelia is indicated on the right. The bootstrap support of each node is provided. The scale bar indicates 10% divergence. **B** Alignment of the Sec7 domains. At the top, the Sec7 domain of human cytohesin-1 is shown. The alignment was generated with the program Multalin (http://bioinfo.genotoul.fr/multalin/multalin.html). The color code and the symbols represent: upper case  =  high homology (red); lower case  =  lower homology (blue); !  =  mostly I, V; $  =  mostly L, M; %  =  mostly F, Y; #  =  mostly D, E, N, Q. The invariable E involved in nucleotide exchange on Arf [8] is indicated by an arrow.

**Figure 2:**
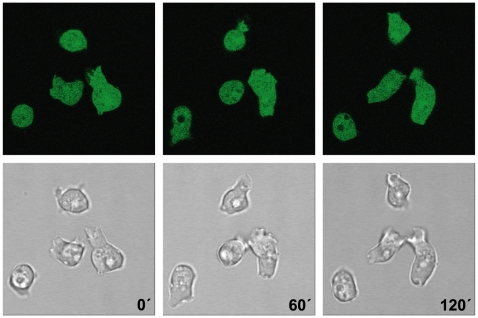
Distribution of GFP-SecG in living cells. Localization of GFP-tagged full length SecG analyzed by live-cell imaging. Three frames taken at the indicated time points after start of the analysis are shown.

A Sec7 domain analysis revealed three groups in the phylogenetic tree of the Sec7 family members. Group 1 clusters with human cytohesin-1 and contains the secG homologues and proteins composed of a Sec7 and PH domain only, group 2 is formed by the BIG family members, and group 3 contains the GBF family proteins and the cytohesin family which is distinguished by the presence of a putative transmembrane region (Fig. 1A, Table 1). Closer inspection of the Sec7 domain specific motifs 1 (position 105–110 in the human cytohesin-1 Sec7 domain, Fig. 1B) and 2 (position 149–162, Fig. 1B) which are required for the nucleotide exchange on Arf shows that they are quite well conserved. Only in the BIG family members DDB0233618, DFA_12170 and PPL_04049 motif 1 is more divergent, however, it should be noted that the invariable glutamic acid (E) involved in nucleotide exchange on Arf is present throughout (arrow in Fig. 1B). [8].

### Generation of a *D. discoideum* SecG Deficient Mutant

**Figure 3:**
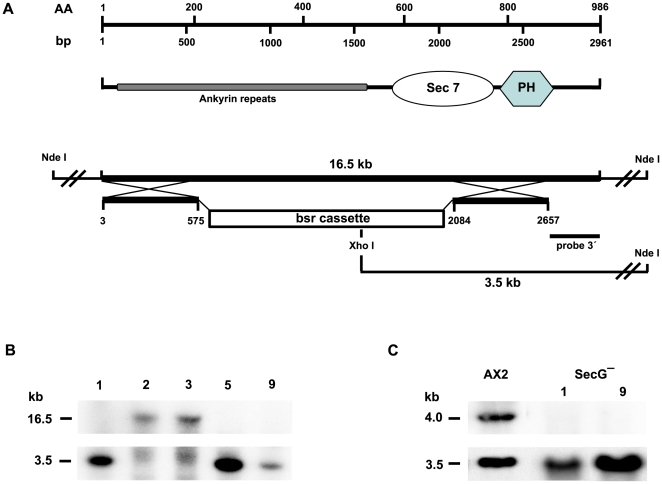
Generation of secG deficient cells. **A**. Upper part: SecG domain structure. The domain structure of Sec G aligned along the nucleotide and amino acid sequence is schematically depicted. **A.** Lower part: Generation of a gene replacement vector. The vector was constructed by replacing an internal segment of the secG gene extending from position 576 to 2083 with the blasticidin resistance cassette (bsr). The secG gene is located on a 16.5 kb NdeI genomic fragment. Location of relevant restriction enzyme sites and of the probe used for Southern blot analysis is given. **B.** Southern blot analysis of XhoI/NdeI digested genomic DNA of individual transformants 1, 2, 3, 5, and 9. After successful replacement a shift from 16.5 kb for the wild type band (transformants 2, 3) down to 3.5 kb (transformants 1, 5, 9) occurred as detected by a 3′ gene specific probe. **C.** Northern blot analysis. In *secG^−^* transformants (1, 9) the ∼4 kb mRNA of AX2 wild type is no longer detected using the 350 bp 3′ probe. For control of loading, the blot was probed with a ddFLN specific probe recognizing the ∼3.5 kb mRNA [45].

The secG gene of *D. discoideum* is located on chromosome 5. It contains no intron and gives rise to a ∼4 kb mRNA present during growth and development. SecG is a 986 aa protein which when expressed as GFP fusion is present throughout the cytosol (Fig. 2). To test a possible association with the cytoskeleton we lysed the cells with Triton X-100 and fractionated the lysate into Triton-insoluble material which represents the cytoskeleton and Triton-soluble material. The GFP-tagged protein remained completely in the supernatant indicating that SecG is primarily a cytosolic protein (data not shown). To investigate the SecG role in adhesion and cell motility we generated SecG deficient cells in strain AX2 using a gene replacement vector in which sequences from position 576 to 2083 of the coding sequence encoding 10 of the predicted 15 ankyrin repeats and the first half of the Sec7 domain (amino acid residues 192 to 695) were replaced by the selection cassette. The replacement event was confirmed by PCR and Southern blot analysis. Northern blot analysis showed the absence of the mRNA (Fig. 3). Growth of *secG^−^* cells in axenic medium and on a lawn of *Klebsiella* was comparable to wild type AX2. Under osmotic stress conditions growth behaviour was also similar to the one of AX2. The mutant strains developed in a timely manner in shaken suspension under starvation conditions as well as on phosphate agar plates and formed normal fruiting bodies (data not shown).

In our further analysis we focused primarily on cell-substrate and cell-cell adhesion and on cell motility as these are aspects in which mammalian cytohesins play a prominent role.

### Cell-Substrate and Cell-Cell Adhesion Characteristics of the *secG^−^* Strains

**Figure 4:**
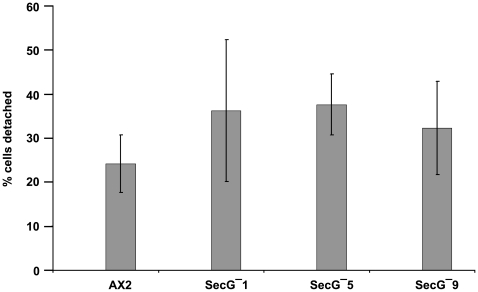
Cell-substratum adhesion in *secG^−^* cells. Adhesion of vegetative cells was measured. After a 4 hour incubation period the cells were subjected to rotation at 65 rpm on a gyratory shaker. The number of detached cells was determined after one hour of shaking and set in relation to the total number of cells. AX2 wild type and three different mutant cell lines were examined. The results are from 23 independent experiments for AX2 and a total of 39 experiments for the mutant cell lines. Shown are the mean values and the mean deviations. The P values (secG-1, 0.0721; secG-5, 0.0903; secG-9; 0.0964) were not quite statistically significant.

To assay cell-substrate adhesion cells are kept in a Petri dish where they settle on the surface. Four hours after start of the experiment we assessed attachment by subjecting the cells to rotation on an orbital shaker at 65 rpm for one hour. Then the number of unattached cells was determined as well as the total number of cells after resuspension of all cells, and the percentage of detached cells was calculated. Approximately 25% of wild type cells were detached from the substratum when shaken at 65 rpm for one hour. For three individual mutant strains we observed 36, ∼38 and 32% of all cells in the supernatant (Fig. 4). Although the standard deviation is rather high in these experiments, the mutant strains are clearly less adhesive than AX2.

Cell-cell adhesion is essential for multicellular development of *Dictyostelium* and several proteins that mediate this adhesion are expressed in an ordered fashion [16]. The strength of the adhesion can be assayed by determining the extent of re-aggregation after dissociation of aggregating cells. We developed cells on phosphate agar plates, harvested them after 10 to 12 hours and mechanically dissociated them into single cells. Then they were incubated with shaking at 60 rpm and the number of single cells was determined after one and two hours. For all strains re-association of the cells into large aggregates was complete after two hours.

### *secG^−^* Cells Do Not Have a Defect in Cell Sorting

**Figure 5:**
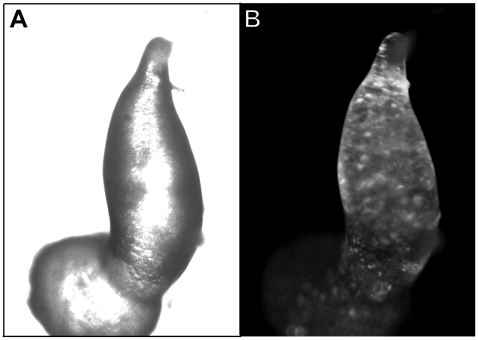
Distribution of labelled AX2 cells in a chimeric developmental organism. AX2 cells expressing GFP-LimD were mixed with *secG^−^* cells in a ratio of 10∶90. The GFP-labelled wild type cells show a random distribution at the multicellular stage (culminant). The phase contrast (**A**) and the fluorescence (**B**) image are shown.

Cell sorting in the mound and slug stage of *Dictyostelium* development is mediated by a combination of differential chemotaxis and cell-cell adhesion. We therefore performed mixing experiments of wild type and mutant cells. These experiments will give further information on cell adhesion during development. We carried out the experiment with *secG^−^* cells expressing GFP-LimD or AX2 cells expressing GFP-LimD. The GFP-expressing cells were mixed in a ratio of 10∶90 with unlabelled wild type or mutant cells as appropriate and allowed to develop on phosphate agar plates. We then analysed the distribution of wild type and mutant cells in the chimeras at the mound and slug stage by microscopic inspection. We did not observe a sorting out of mutant or wild type cells, instead, the fluorescently labelled cells were randomly distributed throughout the mounds and slugs irrespective of their origin and were not specifically enriched in either the prespore or the prestalk region (Fig. 5). The results show that mutant and wild type cells are indistinguishable with regard to their cell surface proteins and do not sort out and also that they develop in a comparable manner.

### Uptake of Bacteria and of Yeast Particles

Bacteria are the favourite food source of *D. discoideum* cells in the wild. Their uptake is most likely initiated by binding to receptors at the membrane and triggering signalling processes required for uptake. The adhesion receptors have however not been identified [17]. We analysed growth on a lawn of *Klebsiella* and growth on *E. coli* B/r in suspension and determined the growth rates of AX2 and the *secG* mutants. All mutant strains grew like AX2 with the bacteria as food source either on agar plates containing *Klebsiella* or in suspension with *E*. *coli* B/r where they had comparable duplication times of approximately three hours. Similarly, in yeast uptake assays where we quantified the ingested particles after 10, 20 and 30 minutes we found that *secG^−^* cells were as efficient in uptake as was AX2. In a representative experiment 3.25% of AX2 cells and 4.7% of *secG^−^* cells had taken up yeast particles after 10 minutes of incubation, after 20 minutes 12.82% of AX2 and 11.33% of *secG^−^* and after 30 minutes 19.92% of AX2 and 19.30% of *secG^−^* had ingested yeast.

### Cell Motility and Chemotaxis in *secG^−^* Cells

*Dictyostelium* cells exhibit an amoeboid type of cell motility. They deform very quickly and translocate via rapidly alternating cycles of pseudopod extension and pseudopod retraction in response to external signals which are dependent on changes in the actin cytoskeleton. Similar amoeboid movements are observed in hematopoietic stem cells, leukocytes and certain tumor cells. In contrast to movement by other mammalian cells such as fibroblasts or keratinocytes they do not depend on β1-integrin-mediated adhesion [18] [19].

**Figure 6:**
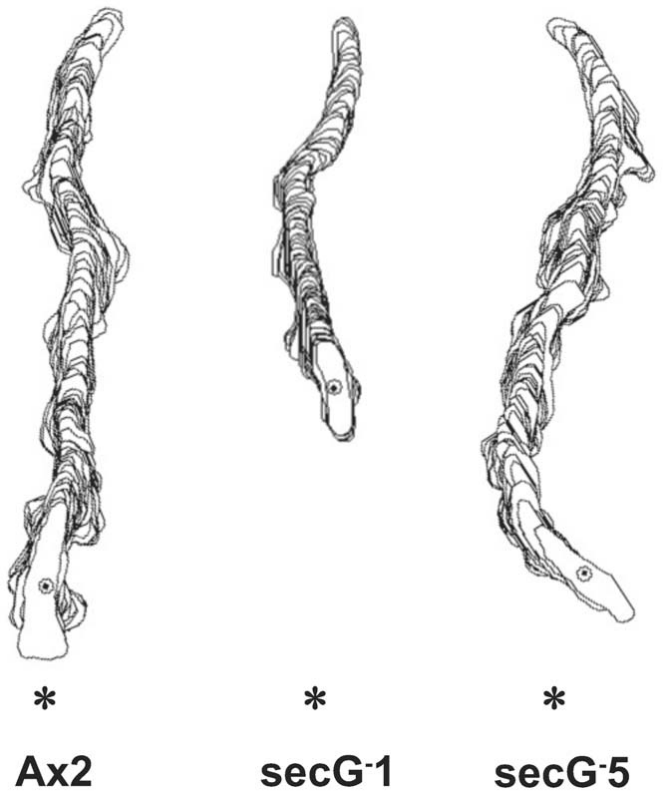
Chemotaxis of aggregation competent cells. AX2 wild type and *secG^−^* cell lines 1 and 5 were subjected to a chemotaxis assay. Cells were traced from the movies and analysed by DIAS software [41]. Representative stacked images are shown. The star indicates the location of the cAMP source.

To determine if *secG^−^* cells exhibit defects in cell motility and chemotaxis during aggregation we examined the parameters of cAMP mediated chemotaxis of individual aggregation competent cells. Mutant cells consistently displayed a reduced speed (∼8 versus ∼10 µm/min for AX2). They were however highly elongated and extended pseudopods mainly in the direction of the chemoattractant. All parameters analysed, directionality, direction change, persistence and roundness, did not differ significantly suggesting that SecG of *D. discoideum* plays no role in chemotaxis but its absence leads to a speed reduction (Table 2, Fig. 6).

## Discussion

Cytohesins are intracellular proteins that can be recruited to the plasma membrane due to binding of their PH domains to phosphatidylinositol phospholipids. At the plasma membrane they interact with integrins and influence cell adhesion and cell migration. Here we studied the role of the *D. discoideum* cytohesin homolog SecG in adhesion and motility in order to unravel the basic roles of cytohesins. Until now cytohesins have been considered metazoan specific. We show that SecG closely resembles cytohesin-1 in its Sec PH tandem domain, further, we identified SecG homologs in *Dicytostelidae* groups that are phylogenetically at the base.

Three forms of adhesion are important for *Dictyostelium* growth and development, adhesion of bacteria to the surface of amoebae during growth phase, cell-substrate adhesion for locomotion when the amoebae chase their prey, during aggregation and at later stages of development, and cell-cell adhesion when the cells undergo multicellular development [16]. The molecular components responsible for cell-cell adhesion have been studied for some time whereas the other types of adhesion have come into the focus of research much more recently.

*Dictyostelium* cells exhibit an amoeboid movement. Such type of movement is characterized by a high membrane turnover which is opposed to strong attachment mediated by focal adhesions. This explains why strong focal adhesions as they exist in mammalian cells are not present. However, cells do exhibit cell-substrate adhesion and molecules mediating them have been identified and characterized such as SadA, a plasma membrane protein with nine putative transmembrane domains and tenascin-like EGF repeats in the extracellular region. SadA deficient cells cannot initiate attachment to a plastic surface nor do they take up latex beads, furthermore vegetative cells move faster than wild type [20]. *Dictyostelium* paxillin might provide a link between components of the plasma membrane and the cytoskeleton similar to mammalian paxillin which is an essential regulatory component of focal adhesions and couples integrins to the actin cytoskeleton [21] [22]. Loss of paxillin in *D. discoideum* leads to reduced cell-substratum adhesion of vegetative cells and impaired multicellular development [23].

Cell-cell adhesion is mediated by developmental stage specific proteins in *Dictyostelium* [24] [25]. They belong to the immunoglobulin superfamily in case of contact site A or represent cadherin type molecules in case of DdCAD-1. Loss of csA did not affect development in the mutant cells under laboratory conditions, however when grown on soil plates which resembles natural conditions the mutant cells displayed reduced cell-cell adhesion, increased adhesiveness to the substratum, and slower motility, which led to their sorting out from aggregating wild-type cells [26] [27]. Loss of DdCAD-1 resulted in abnormal slug morphology and a delay in culmination [28]. Adhesion regulators are Rap1 and its activators (RapGAPs) which are important for differential adhesion during development, cell type patterning and morphogenesis [29] [30].

Based on SecG homology to mammalian cytohesins we probed its roles in cell-cell, cell-substrate adhesion and cell motility. Like the cytohesins it is a cytosolic protein which has the potential to associate with lipids through its PH domain. In the cytohesins the Sec7 domain can associate with integrins. Adhesion molecules with homologies to integrins have been described only recently in *D. discoideum* [31] [32]. They were designated Sib, similar to integrins, based on structural and functional homology to mammalian integrins and are potential interactors of the cytohesin-like proteins. SibA, one of the three family members, interacts with *Dictyostelium* talin. Talin is also a well known partner of mammalian integrins. Inactivation of the sibA gene affected adhesion to phagocytic particles, cell-cell and cell-substrate adhesion. Similarly, talin null cells of *Dictyostelium* have an adhesion and phagocytosis defect [33].

When we compare the phenotypes reported for the SadA, SibA, talin, paxillin, Rap and RapGAPs with the one of *secG^−^*, we find that the phenotype of the secG mutant is less complex and we cannot easily link SecG to any of theses proteins. The main defect is in cell-substrate adhesion during growth. Adhesion to bacteria was not affected neither was cell-cell adhesion during development. The cells were chemotactically active during development, they acquired a polarized cell shape and migrated in a directed fashion towards the chemotactic agent whereas speed was slightly reduced. We conclude that SecG primarily acts in cell-substrate adhesion. Other types of adhesion may be ensured by different proteins. As cellular behaviour is often multiply guaranteed, it might well be that the closely related Sec7-PH domain protein DDB0233617 (DDB_G0272486) acts in similar pathways as SecG and takes over some of its functions in the mutant [34].

## Materials and Methods

### *Dictyostelium* Strains and Mutant Generation

*D. discoideum* strain AX2 was used as the parental strain. Growth and development were done as described [35]. For generation of secG deficient cells a knockout vector was generated by amplifying DNA sequences of the intron-less gene (DDB_G0287459) from positions 3–575 and 2084–2657 of the coding sequence and cloning them into the Cre-*loxP* vector pLPBLP carrying a blasticidin resistance conferring gene [36]. The plasmid was linearized and transformed into AX2 cells. Transformants were selected using Blasticidin S (MP Biomedicals, Eschwege, Germany) at 1.5 µg/ml and analysed by PCR and Southern blot. Three mutant cell lines, *segG^−^* 1, 5 and 9 were analysed giving identical results. A plasmid allowing expression of GFP-SecG was obtained from Dr. D. Brazil [37]. Expression was under the control of the actin15 promoter. An ethics statement is not required for this work.

### Mutant Analysis

Strains were analysed for growth in axenic medium both under standard conditions as well as under osmotic stress conditions by addition of 115 mM sorbitol or 30 mM NaCl. Growth was also analysed on bacterial plates using *Klebsiella* as food source. Development was assayed during starvation in suspension (in Soerensen phosphate buffer, 2 mM Na_2_HPO_4_, 14.6 mM KH_2_PO_4_, pH 6.0) and on phosphate agar plates.

### Adhesion Assays

To analyse cell-substrate adhesion a substrate detachment assay was carried out [20]. A total of 1×10^6^ cells in growth medium was added per well (24 well plates, Costar) and incubated for four hours at 22°C. Then the plates were shaken on a gyrotary shaker at 65 rpm for one hour. The number of detached cells was counted in a hemocytometer. The total number of cells was determined after resuspension of all cells and the percentage of detached cells calculated. To analyse cell-cell adhesion during development [29] cells were starved on phosphate agar plates and harvested after 10 to 12 hours once they had formed tight aggregates. The cells in the aggregates were dissociated by repeated passage through a 21G needle, 1×10^6^ cells in Soerensen phosphate buffer were added per well (24 well plate) and shaken at 60 rpm. The number of single cells was determined one and two hours after the start of shaking.

### Cell Mixing Experiments

For examining the distribution of the cells in a chimeric developmental organism, 10% GFP-LimD [35] expressing AX2 wild-type cells were mixed with 90% unlabeled *secG^−^* cells or 10% GFP-LimD expressing *secG^−^* cells with 90% unlabeled AX2 cells and developed on phosphate agar plates. The images were collected on a microscope (Leica) with DIC and fluorescence imaging.

### Growth on Bacteria and Uptake of Yeast Particles

*E. coli* B/r was grown to saturation, the cells were pelleted and washed with Soerensen phosphate buffer and concentrated to a density of 1×10^10^ cells per ml. *Dictyostelium* cells growing in suspension were harvested, washed, resuspended in Soerensen phosphate buffer and used at a density of 1×10^5^ cells/ml for inoculation of the *E. coli* B/r suspension. Shaking was at 160 rpm. The increase in cell number was determined every two hours. Yeast phagocytosis was analysed on a substratum as described [38].

### Analysis of Chemotaxis

Cells starved for 5 h were used for chemotaxis assays [39]. They were stimulated with a glass capillary micropipette (Eppendorf Femtotip) filled with 0.1 mM cAMP, which was attached to a microcontroller. Time-lapse image series were captured and stored on a computer hard drive at 30 seconds intervals with a JAI CV-M10 CCD camera and an Imagenation PX610 frame grabber (Imagenation Corp., Beaverton, OR) controlled through Optimas software (Optimas Corp., Bothell, Washington). The DIAS software (Solltech, Oakdale, IA) was used to trace individual cells along image series and automatically outlined the cell perimeters and converted them to replacement images from which the position of the cell centroid was determined [40]. Speed and change of direction were computed from the centroid position [41]. For processing images, Corel Draw version 11 and Adobe Photoshop were used. Chemotaxing GFP-CRN7 expressing cells were observed under a cAMP gradient in a chemotaxis chamber (μ-slide chemotaxis hydrophobic, uncoated, and sterile, ibidi, Martinsried, Germany).

### Miscellaneous Methods

Immunofluorescence analysis was done as described [42]. Actin was detected with mAb Act-1–7 [43] followed by incubation with Cy3-labeled goat anti-mouse IgG secondary antibody. Confocal microscopy was done using a Leica TCS-SP5 confocal laser scanning microscope [44]. Distribution and live dynamics of GFP-SecG were analyzed. Images were processed using the accompanying Leica software or Image J. The preparation of TritonX-100 insoluble cytoskeletons is described in [45].

## References

[1] Arnaout MA, Goodman SL, Xiong JP (2007). Structure and mechanics of integrin-based cell adhesion.. Curr Opin Cell Biol.

[2] Quast T, Tappertzhofen B, Schild C, Grell J, Czeloth N (2009). Cytohesin-1 controls the activation of RhoA and modulates integrin-dependent adhesion and migration of dendritic cells.. Blood.

[3] Kolanus W, Nagel W, Schiller B, Zeitlmann L, Godar S (1996). Alpha L beta 2 integrin/LFA-1 binding to ICAM-1 induced by cytohesin-1, a cytoplasmic regulatory molecule.. Cell.

[4] D'Souza-Schorey C, Chavrier P (2006). ARF proteins: roles in membrane traffic and beyond.. Nat Rev Mol Cell Biol.

[5] Myers KR, Casanova JE (2008). Regulation of actin cytoskeleton dynamics by Arf-family GTPases.. Trends Cell Biol.

[6] Gillingham AK, Munro S (2007). Identification of a guanine nucleotide exchange factor for Arf3, the yeast orthologue of mammalian Arf6.. PLoS One.

[7] Cox R, Mason-Gamer RJ, Jackson CL, Segev N (2004). Phylogenetic analysis of Sec7-domain-containing Arf nucleotide exchangers.. Mol Biol Cell.

[8] Cherfils J, Ménétrey J, Mathieu M, Le Bras G, Robineau S (1998). Structure of the Sec7 domain of the Arf exchange factor ARNO.. Nature.

[9] Béraud-Dufour S, Robineau S, Chardin P, Paris S, Chabre M (1998). A glutamic finger in the guanine nucleotide exchange factor ARNO displaces Mg2+ and the beta-phosphate to destabilize GDP on ARF1.. EMBO J.

[10] DiNitto JP, Delprato A, Gabe Lee MT, Cronin TC, Huang S (2007). Structural basis and mechanism of autoregulation in 3-phosphoinositide-dependent Grp1 family Arf GTPase exchange factors.. Mol Cell.

[11] Eichinger L, Pachebat JA, Glöckner G, Rajandream M.-A, Sucgang R (2005). The genome of the social amoeba *Dictyostelium discoideum*.. Nature.

[12] Schaap P (2007). Evolution of size and pattern in the social amoebas.. Bioessays.

[13] Bloomfield G, Tanaka Y, Skelton J, Ivens A, Kay RR (2008). Widespread duplications in the genomes of laboratory stocks of *Dictyostelium discoideum*.. Genome Biol.

[14] Mouratou B, Biou V, Joubert A, Cohen J, Shields DJ (2005). The domain architecture of large guanine nucleotide exchange factors for the small GTP-binding protein Arf.. BMC Genomics.

[15] Kolanus W (2007). Guanine nucleotide exchange factors of the cytohesin family and their roles in signal transduction.. Immunol Rev.

[16] Bozzaro S, Ponte E (1995). Cell adhesion in the life cycle of *Dictyostelium*.. Experientia.

[17] Sillo A, Bloomfield G, Balest A, Balbo A, Pergolizzi B (2008). Genome-wide transcriptional changes induced by phagocytosis or growth on bacteria in *Dictyostelium*.. BMC Genomics.

[18] Wolf K, Müller R, Borgmann S, Bröcker EB, Friedl P (2003). Amoeboid shape change and contact guidance: T-lymphocyte crawling through fibrillar collagen is independent of matrix remodeling by MMPs and other proteases.. Blood.

[19] Friedl P (2004). Prespecification and plasticity: shifting mechanisms of cell migration.. Curr Opin Cell Biol.

[20] Fey P, Stephens S, Titus MA, Chisholm RL (2002). SadA, a novel adhesion receptor in *Dictyostelium*.. J Cell Biol.

[21] Crowley E, Horwitz AF (1995). Tyrosine phosphorylation and cytoskeletal tension regulate the release of fibroblast adhesions.. J Cell Biol.

[22] Norman JC, Jones D, Barry ST, Holt MR, Cockcroft S (1998). ARF1 mediates paxillin recruitment to focal adhesions and potentiates Rho-stimulated stress fiber formation in intact and permeabilized Swiss 3T3 fibroblasts.. J Cell Biol.

[23] Bukharova T, Weijer G, Bosgraaf L, Dormann D, van Haastert PJ (2005). Paxillin is required for cell-substrate adhesion, cell sorting and slug migration during *Dictyostelium* development.. J Cell Sci.

[24] Müller K, Gerisch G, Fromme I, Mayer H, Tsugita A (1979). A membrane glycoprotein of aggregating *Dictyostelium* cells with the properties of contact sites.. Eur J Biochem.

[25] Wong EF, Brar SK, Sesaki H, Yang C, Siu CH (1996). Molecular cloning and characterization of DdCAD-1, a Ca2+-dependent cell-cell adhesion molecule, in *Dictyostelium discoideum*.. J Biol Chem.

[26] Harloff C, Gerisch G, Noegel AA (1989). Selective elimination of the contact site A protein of Dictyostelium discoideum by gene disruption. Genes Dev.

[27] Ponte E, Bracco E, Faix J, Bozzaro S (1998). Detection of subtle phenotypes: the case of the cell adhesion molecule csA in *Dictyostelium*.. Proc Natl Acad Sci U S A.

[28] Wong E, Yang C, Wang J, Fuller D, Loomis WF (2002). Disruption of the gene encoding the cell adhesion molecule DdCAD-1 leads to aberrant cell sorting and cell-type proportioning during *Dictyostelium* development.. Development.

[29] Parkinson K, Bolourani P, Traynor D, Aldren NL, Kay RR (2009). Regulation of Rap1 activity is required for differential adhesion, cell-type patterning and morphogenesis in *Dictyostelium*.. J Cell Sci.

[30] Jeon TJ, Lee S, Weeks G, Firtel RA (2009). Regulation of *Dictyostelium* morphogenesis by RapGAP3.. Dev Biol.

[31] Cornillon S, Gebbie L, Benghezal M, Nair P, Keller S (2006). An adhesion molecule in free-living *Dictyostelium* amoebae with integrin beta features.. EMBO Rep.

[32] Cornillon S, Froquet R, Cosson P (2008). Involvement of Sib proteins in the regulation of cellular adhesion in *Dictyostelium discoideum*.. Eukaryot Cell.

[33] Niewöhner J, Weber I, Maniak M, Müller-Taubenberger A, Gerisch G (1997). Talin-null cells of *Dictyostelium* are strongly defective in adhesion to particle and substrate surfaces and slightly impaired in cytokinesis.. J Cell Biol.

[34] Witke W, Schleicher M, Noegel AA (1992). Redundancy in the microfilament system: abnormal development of *Dictyostelium* cells lacking two F-actin cross-linking proteins.. Cell.

[35] Khurana B, Khurana T, Khaire N, Noegel AA (2002). Functions of LIM proteins in cell polarity and chemotactic motility.. EMBO J.

[36] Faix J, Kreppel L, Shaulsky G, Schleicher M, Kimmel A (2004). A rapid and efficient method to generate multiple gene disruptions in *Dictyostelium discoideum* using a single selectable marker and the Cre-loxP system.. Nucleic Acids Res.

[37] Thomason PA, Brazill DT, Cox EC (2006). A series of *Dictyostelium* expression vectors for recombination cloning.. Plasmid.

[38] Schreiner T, Mohrs MR, Blau-Wasser R, von Krempelhuber A, Steinert M (2002). Loss of the F-actin binding and vesicle associated protein comitin leads to a phagocytosis defect.. Eukaryot Cell.

[39] Gerisch G, Keller HU (1981). Chemotactic reorientation of granulocytes stimulated with micropipettes containing fMet-Leu-Phe.. J Cell Sci.

[40] Noegel AA, Blau-Wasser R, Sultana H, Israel L, Müller R (2004). CAP/ASP56 as regulator of cell polarity and cAMP signaling in *Dictyostelium*.. Mol Biol Cell.

[41] Wessels D, Voss E, Von Bergen N, Burns R, Stites J (1998). A computer-assisted system for reconstructing and interpreting the dynamic three-dimensional relationships of the outer surface, nucleus and pseudopods of crawling cells.. Cell Motil Cytoskeleton.

[42] Weiner OH, Murphy J, Griffiths G, Schleicher M, Noegel AA (1993). The actin-binding protein comitin (p24) is a component of the Golgi apparatus.. J Cell Biol.

[43] Simpson PA, Spudich JA, Parham P (1984). Monoclonal antibodies prepared against *Dictyostelium* actin: characterization and interactions with actin.. J Cell Biol.

[44] Knuth M, Khaire N, Kuspa A, Lu SJ, Schleicher M (2004). A novel partner for *Dictyostelium* filamin is an *α*-helical, developmentally regulated protein.. J Cell Sci.

[45] Brink M, Gerisch G, Isenberg G, Noegel AA, Segall JE (1990). A *Dictyostelium* mutant lacking an F-actin crosslinking protein, the 120 kD gelation factor.. J Cell Biol.

[46] Casanova JE (2007). Regulation of Arf activation: the Sec7 family of guanine nucleotide exchange factors.. Traffic.

